# The paradigm-shifting idea and its practice: from traditional abortion Chinese medicine *Murraya paniculata* to safe and effective cancer metastatic chemopreventives

**DOI:** 10.18632/oncotarget.7932

**Published:** 2016-03-05

**Authors:** Zhou Jiang, Yaqiong Pang, Xiaobo Yu, Suxia Zhou, Jun Qian, Ning Zheng, Haiyan Dong, Qing Shi, Minliang Kuo, Lee Jia

**Affiliations:** ^1^ Cancer Metastasis Alert and Prevention Center, and Biopharmaceutical Photocatalysis, State Key Laboratory of Photocatalysis on Energy and Environment, Fuzhou University, Fuzhou, China; ^2^ Fujian Provincial Key Laboratory of Cancer Metastasis Chemoprevention and Chemotherapy, Fuzhou University, Fuzhou, China; ^3^ Graduate Institute of Biomedical Sciences, College of Life Science, National Taiwan University, Taipei, Taiwan

**Keywords:** cancer metastatic chemoprevention, circulating tumor cells, cell adhesion molecules, traditional abortion Chinese medicine, Murraya paniculata

## Abstract

Recent large epidemiological studies demonstrated benefit of oral contraceptives in reducing cancer risk, and our analysis also showed molecular and cellular similarities between embryo implantation and CTCs adhesion-invasion to endothelium. We here hypothesize that abortion traditional Chinese medicine (TCM) may serve well for pre-metastatic chemoprevention. To test the hypothesis, we selected the safe and well-known abortifacient TCM *Murraya paniculata* and identified a most-promising extracted fraction G (containing flavonoids and coumarins) from its many raw ethanol/dichloromethane extracts by using the bioactivity-guided fast screen assay. G showed free radical scavenging effect, and specifically inhibited both embryo implantation to human endometrial bed and cancer HT29 cells to human endothelium in a concentration-dependent manner (1–30 μg/mL) without significant cytotoxicity demonstrated by its high adhesion inhibition ratio. The inhibition may result from its down-regulation on expression of integrin *β*1 and *α*6, and CD44 on HT29 cells, as well as E-selectin on endothelial cells. Furthermore, G inhibited invasion and migration of HT29 cells. Pretreatment followed by one-month oral administration of G to the immunocompetent mice inoculated with mouse melanoma cells produced significant inhibition on lung metastasis without marked side effects. Collectively, this paradigm-shifting study provides, for the first time, a new strategy to discover safe and effective pre-metastatic chemopreventives from abortion TCM.

## INTRODUCTION

Cancer metastasis is estimated to be responsible for approximately 90% of all cancer deaths and this situation has changed a little over the last eighty years [1–7]. On the other hand, there were nearly 14.5 million cancer survivors in the USA alone on January 1, 2014, and the number will increase to nearly 19 million by 2024 [3]. The difference between the increasing number of cancer survivors after primary cancer treatment and the almost unchanged number of cancer death over decades indicates that the majority of cancer survivors die of cancer metastases. Although many factors can be blamed, global ignorance of cancer pre-metastasis chemoprevention plays the main role. Today, there is an urgent need to establish a cancer metastasis prevention program to find safe and efficient cancer metastasis preventive agents for cancer survivors to cope with long-term prevention and psychological fear of metastasis, and we are doing just that [8–12].

Metastasis involves a multistep process. Briefly, the circulating tumor cells (CTCs, the root cause of cancer metastasis) are activated by inflammatory factors (for example, chemokines), and if the surrounding microenvironment (pH, pO_2_ and platelets) favors, they will adhere to and interact with capillary endothelial bed, and then extravasate to establish the secondary tumors at sites distant from the primary tumor [6]. With the current understanding of CTCs biology and their metastatic tropism [5, 6, 13], we proposed that targeting the earlier “up-stream” metastatic cascade may be more effective in preventing metastasis than inhibiting the late stage of metastatic events. Recently, we have successfully isolated and sorted the alive CTCs from colorectal cancer patients by using immunomagnetic negative enrichment coupled with flow cytometry [14]. We further demonstrated that the nanomaterial dendrimers coated with dual antibodies against CTCs surface biomarkers [15–17] could specifically capture CTCs and restrain their activity without any cytotoxic effects. A nitric oxide donor compound [8], by directly producing vasorelaxation and interfering with hetero-adhesion of cancer cells to vascular endothelium *via* down-regulating expression of cell adhesion molecules (CAMs) could inhibit CTCs-initiated metastasis cascade. Very recently, we showed that the old drugs aspirin, lysine, mifepristone and doxycycline combined could effectively and safely prevent and treat cancer metastasis by intervening the “seeds” from gemmating on the “soil”, and also strengthening the “soil”. Meanwhile, we demonstrated that some natural products or derivatives [18–20] could safely and effectively prevent cancer metastasis in experimental models.

In 2008 and 2010, *Lancet* and BMJ published [21, 22], respectively, the large epidemiological studies, indicating that long-term administration of oral contraceptives may reduce the risk and mortality of cancers. These studies involved tens of thousands of women from 45 epidemiological studies in 21 countries with malignant epithelial or non-epithelial ovarian cancers, and demonstrated that women who had used oral contraceptives had lower rates of death from all cancers including large bowel, rectum, uterine body, ovarian and main gynecological cancers combined, as well as the circulation diseases. In short, the most important finding of the study is that the longer those women had used oral contraceptives, the greater the reduction in ovarian cancer risk. The overall relative risk decreased by 20% for each 5 years of use. In women who had used oral contraceptives for about 15 years the risk of ovarian cancer was halved. Inspired by these large epidemiological results, we recently analyzed the molecular and cellular similarities and differences between embryo implantation to uterine endometrium and CTCs adhesion to vascular endothelium [23], and found that many molecules, including ICAM, VCAM, selectin, integrin, hormones, Sialyl lewis X, and MMP, are shared by both the embryo implantation and cancer cell adhesion-invasion systems. The analysis led us to investigating the chemopreventive effect of the abortifacients metapristone and mifepristone (RU486) on cancer metastatic models [9, 24–26]. Indeed, the abortifacients showed a good activity at inhibiting cancer metastasis in the *in vitro* and *in vivo* settings.

The cellular and molecular similarities between trophoblastic adhesion and cancer cell adhesion [26–31] tempted us to the traditional abortion Chinese medicinal plants or herbs to look for potential safe and effective metastatic chemopreventives. In the huge treasure, we hunted for the traditional Chinese medicine (TCM) that should have the following properties: safe, abortion, anti-inflammation, anti-coagulation, analgesic, and vasodilation. The TCM *Murraya paniculata* (L.) Jack meets the criteria: It is very safe with the oral LD_50_ value to mice > 5 g/kg (the maximum mouse stomach volume) [32]. Its stem bark extract had been used in China for abortion. It has vasodilation activity [33], anti-inflammatory and anti-coagulation [34], which together disfavor adhesion-invasion of CTCs to the metastatic foci. Considering that TCM is always taken as a raw extract in which various effective components work together to recover the body's Ying-Yang balance [35], the present study will use the raw extract of *Murraya paniculata* (L.) to test its metastatic chemopreventive effects and mechanisms of actions. The paradigm-shifting study emphasizes the relative safety as the foremost criterion in screening the metastatic chemopreventive TCMs, and created the adhesion inhibition ratio (AIR) to distinguish the metastatic chemopreventives from cytotoxic agents.

## RESULTS

### Bioactivity-guided fast screen of active fractions

**Figure 1:**
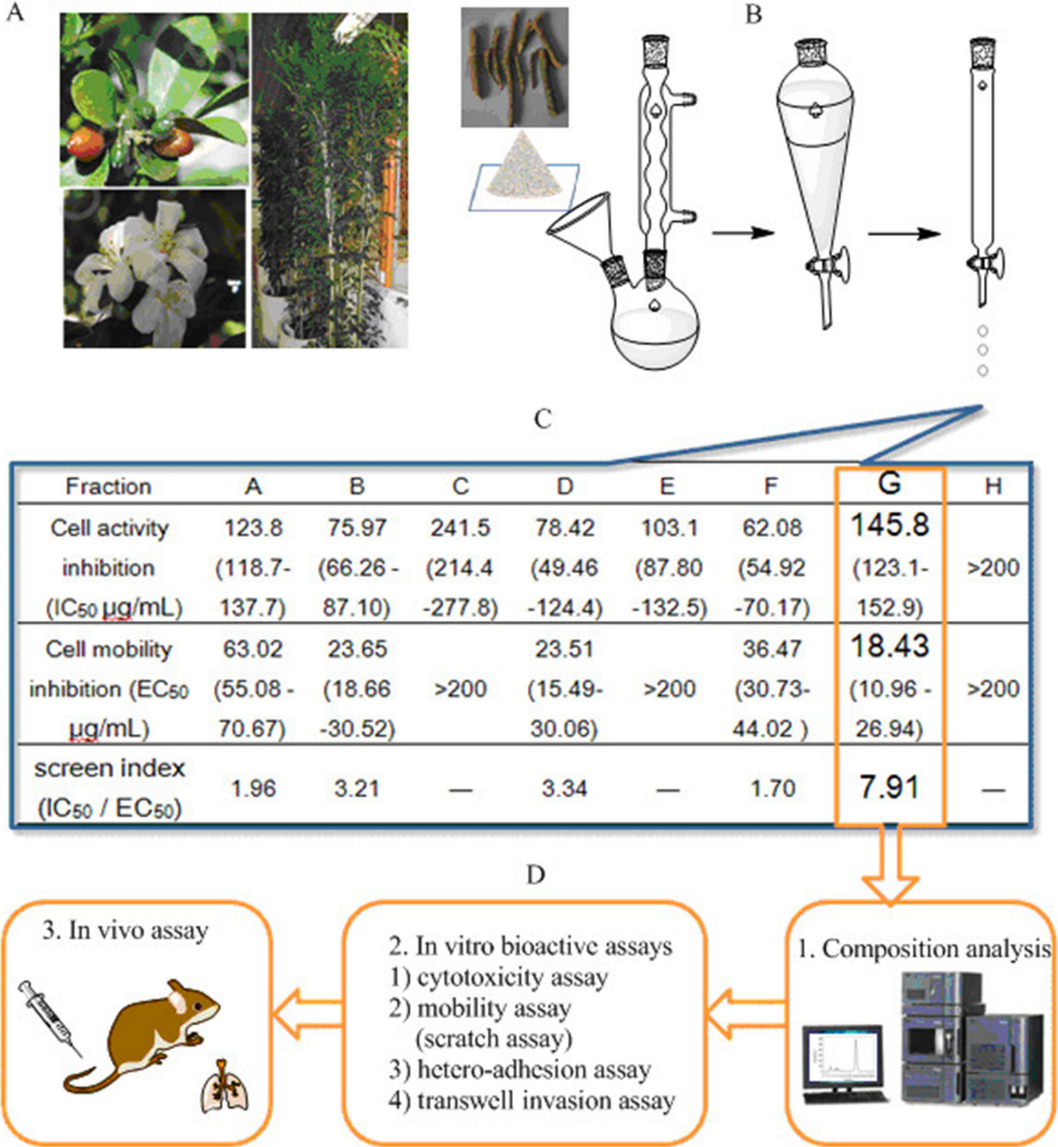
Schematic illustration of bioactivity-guided fast screen for cancer metastatic chemopreventive materials from raw extracts of *M. paniculata.* (**A**) Photographs of typical *M. paniculata*. (**B**) Procedures of extracting bioactive materials from the plant twigs: the twigs were collected and smashed to pieces, and immersed and refluxed with 75% ethanol overnight. The concentrated residual was extracted with dichloromethane, which was concentrated and subjected to gradient silica chromatographic separation. (**C**) Bioactivity-guided fast screen for active raw materials: the concentrated eluates (fractions A-H) were diluted with cell medium, and tested for their inhibition against cancer cell viability (IC_50_) and mobility (EC_50_). The fast screen index was calculated by dividing IC_50_ by EC_50_ of each eluted fraction. The fraction with the highest index was selected, i.e., G, as the best candidate fraction for further composition analysis and *in vitro* and *in vivo* tests (**D**).

The twigs of *M. paniculata* (Figure 1A) were extracted with 75% refluxing ethanol, followed by extraction with dichloromethane to isolate the middle polarity compositions. The dried dichloromethane layer was pooled (∼40 g) and subjected to gradient chromatography. The separation was carried out on a silica gel column with elution of different ratios of acetic ether (EtOAc): petroleum ether (Figure 1B). The resulting eight fractions (labeled from A to H) were obtained and grouped based on their thin layer chromatographic (TLC) similarity. The total extract yield of fraction A to H ranged from 0.017 to 0.09% (w/w), respectively. To quickly identify the interesting fraction that may be potent in inhibiting the cellular activation-adhesion-metastasis process with relative low toxicity, we carried out cell culture screening *in vitro* on HT29 cell line as we described previously [8, 15, 19, 20]. The inhibitory IC_50_ values (the mean drug concentration causing 50% relative growth inhibition of the cells) of each fraction on HT29 cell proliferation were listed on (Figure 1C) based on the concentration-cell activity test results (1–200 μg/mL after 24-h treatment). The fraction G showed the lowest cytotoxicity with IC_50_ value around 145 μg/mL. Completely different from the traditional anticancer drug screen criteria based on the cytotoxicity index of the test drugs, we created, for the first time, the fast screen index to guide our early screen. The screen index was derived from the ratio of IC_50_ to EC_50_ (the mean drug concentration causing 50% relative inhibition of the cell mobility by scratch assay). The higher the screen index is, the more likely the fraction acts as a safe and effective inhibitor that specifically intervenes CTCs adhesion-invasion-metastasis cascade. As shown in Figure 1C, fraction G showed the highest IC_50_ and the lowest EC_50_, and thus the highest screen index (7.91), about 2–3 folds higher than other fractions. As a result, G was selected for more detailed phytochemical and molecular pharmacological analyses (Figure 1D), including chromatographic separation, characterization, and *in vitro* and *in vivo* tests.

### Phytochemical analysis of fraction G

**Figure 2:**
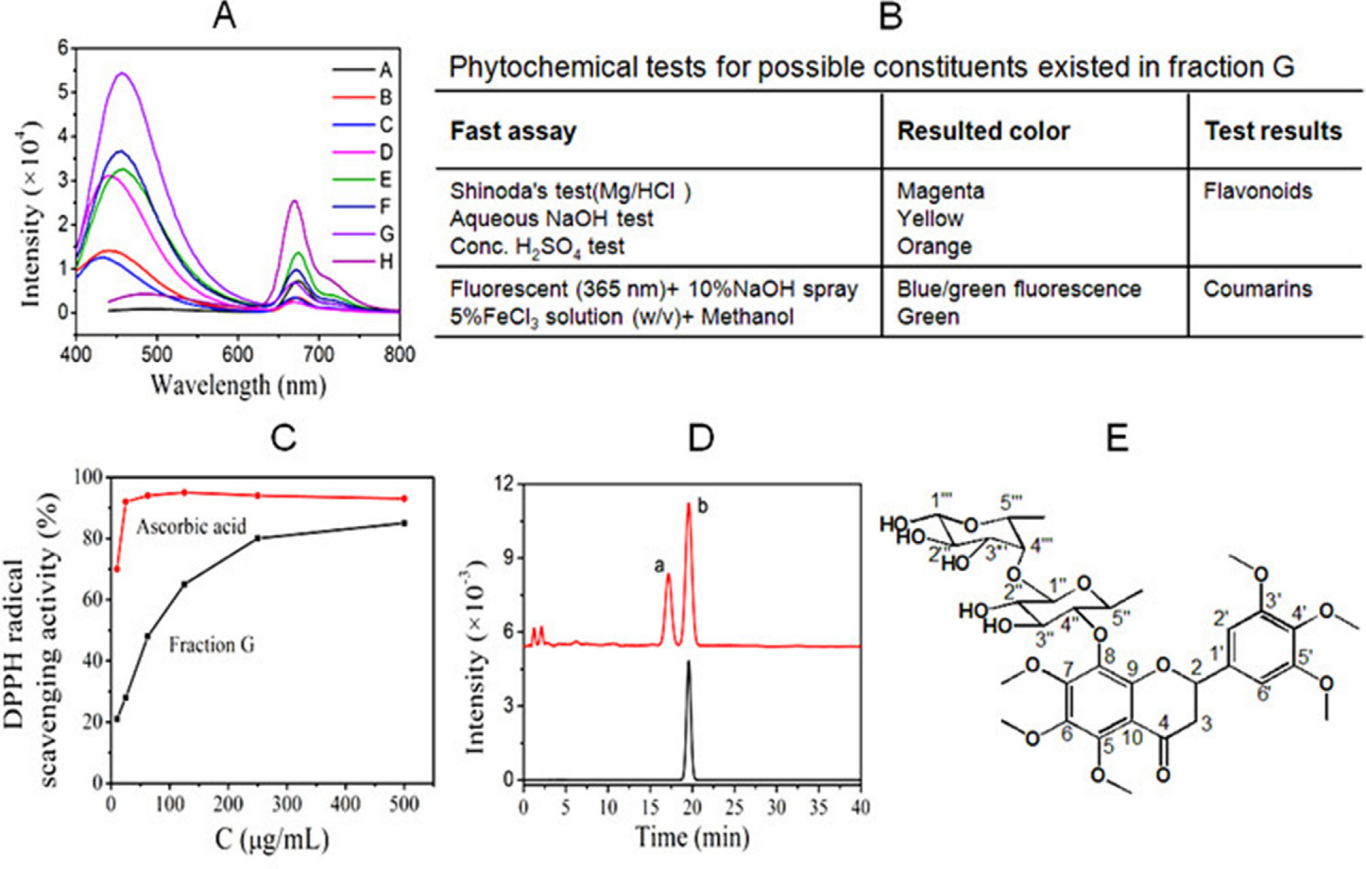
Phytochemical analysis of main components in fraction G following chromatographic separation (**A**) Fluorescent characteristics of *M. paniculata*. dichloromethane-extracted fractions A-H; Note, the G fraction showed the highest fluorescent intensity. (**B**) Phytochemical color tests for possible constituents existed in fraction G; (**C**) DPPH free radical scavenging effect of G in comparison with ascorbic acid; (**D)** HPLC analysis showed two main components (a and b), in fraction G, and semi-preparative HPLC resulted b with satisfying purity. (**E**) Further structural analysis indicated the chemical structure of component b to be HMFRR.

The dichloromethane-extracted fractions of *M. paniculata* were dissolved in methanol, and scanned for fluorescence spectra. G showed the highest fluorescence intensity (Figure 2A). Phytochemical colorimetric tests indicated that G contained both flavonoids and coumarins (Figure 2B). DPPH is the commonly-used agent for quantitatively determining the capacity of the test sample in scavenging free radicals. In comparison with ascorbic acid as the positive control, Figure 2C showed that G contained components that had capacity of scavenging free radicals. HPLC analysis showed two major components (a and b) existed in G, one of which (b) was then purified as a single compound with high purity (Figure 2D) by semi-reparative HPLC. Its structure was elucidated based on ^1^H and ^13^C NMR spectroscopy, and assumed as hexamethoxy flavanone-o-[rhamnopyranosyl–(1→4)-rhamnopyranoside (HMFRR) with the molecular formula of C_33_H_44_O_17_ (Figure 2E). Reported below are ^13^C-NMR and ^1^H-NMR data of HMFRR, which verify its structure: ^13^C-NMR (100 MHz, *d6*-DMSO): *δ* 188.6 (C-4), 162.03 (C-3′), 161.01 (C-5′), 158.81 (C-5), 157.73 (C-7), 156.01 (C-9), 155.51 (C-6), 154.18 (C-4′), 153.39 (C-1′), 139.46 (C-8), 137.8 (C-10), 135.2 (C-6′), 130.5 (C- 2′), 110.58 (C-4′′), 108.56 (C-1′′′), 105.95 (C-4′′′), 104.28 (C- 5′′), 92.06 (C-2′′), 90.76 (C-1′′), 78.94 (C-3′′′), 76.83 (C-3′′), 56.40 (OCH_3_), 56.44 (OCH_3_), 56.61 (OCH_3_), 56.74 (OCH_3_), 56.80 (OCH_3_), 72.39 (C-2), 60.84 (C-2′′′), 60.47 (C-5′′′), 45.31 (C-3), 26.05 (CH_3_ of rhamnose), 25.09 (CH3 of rhamnose). ^1^H-NMR (400 MHz, *d6*-DMSO): *δ* 8.01 (1H, OH-1′′′), 6.85 (4H, OH on rhamnose); 6.64 (2H, H-2′ and H-6′), 6.36 (2H, rhamnose ring), 6.13–6.15 (1H, H-2), 5.42–5.46 (2H, H-3), 4.1 (1H, rhamnose ring), 3.95 (3H, OCH_3_), 3.91 (3H, OCH_3_), 3.82 (3H, OCH_3_), 3.79 (3H, OCH_3_), 3.67 (3H, OCH_3_), 3.65 (3H, OCH_3_), 3.04–3.12 (2H, rhamnose ring), 2.66–2.78 (5H, rhamnose ring), 1.24 (3H, CH_3_ of rhamnose C-5′′), 1.12 (3H, CH_3_ of rhamnose C-5′′′).

### Inhibition of G on biomimetic embryo implantation to endometrium

**Figure 3:**
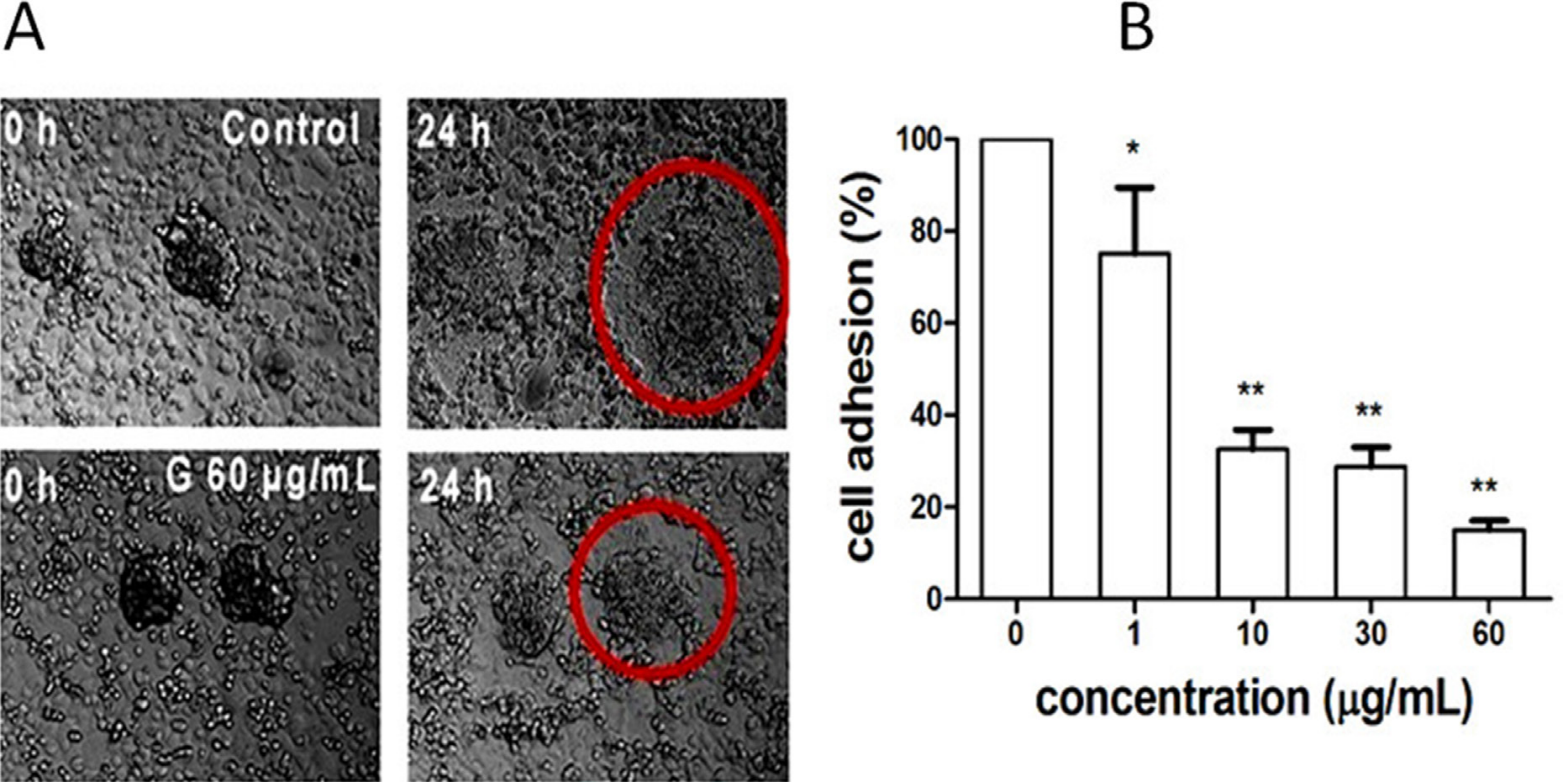
Inhibition of fraction G extracted from *M. paniculata*on **biomimetic embryo implantation to endometrium.** (**A**) Laser confocal microscopy imaging showed the significant inhibition of fraction G (60 μg/mL) on spheroid outgrowth of human placental choriocarcinoma cells JEG-3 spheroids co-cultured with confluent monolayered human endometrial cells RL95-2 for 24 h. (**B**) Concentration–dependent inhibition of fraction G on embryo implantation (spheroid outgrowth of JEG-3 cells to RL95-2 cells). *P < 0.05, and **P < 0.01, compared with the control.

Soon after trophoblast spheroids were formed, we added the spheroids to the confluent monolayer of human endometrial cells in the presence of G (0, 1, 10, 30 and 60 μg/mL). Twenty-four hours later, G showed, in a concentration-dependent manner, significant inhibition on adhesion of the human JEG-3 spheroids to the human endometrial monolayer (Figure 3). The significant inhibition started at 1 μg/mL of G, which was about 145-fold lesser than its inhibition on cell viability (IC_50_= 145 μg/mL; Figure 1C), suggesting the specificity of G on inhibition of embryo implantation to endometrium.

### Effect of G on cancer cells HT29

**Figure 4:**
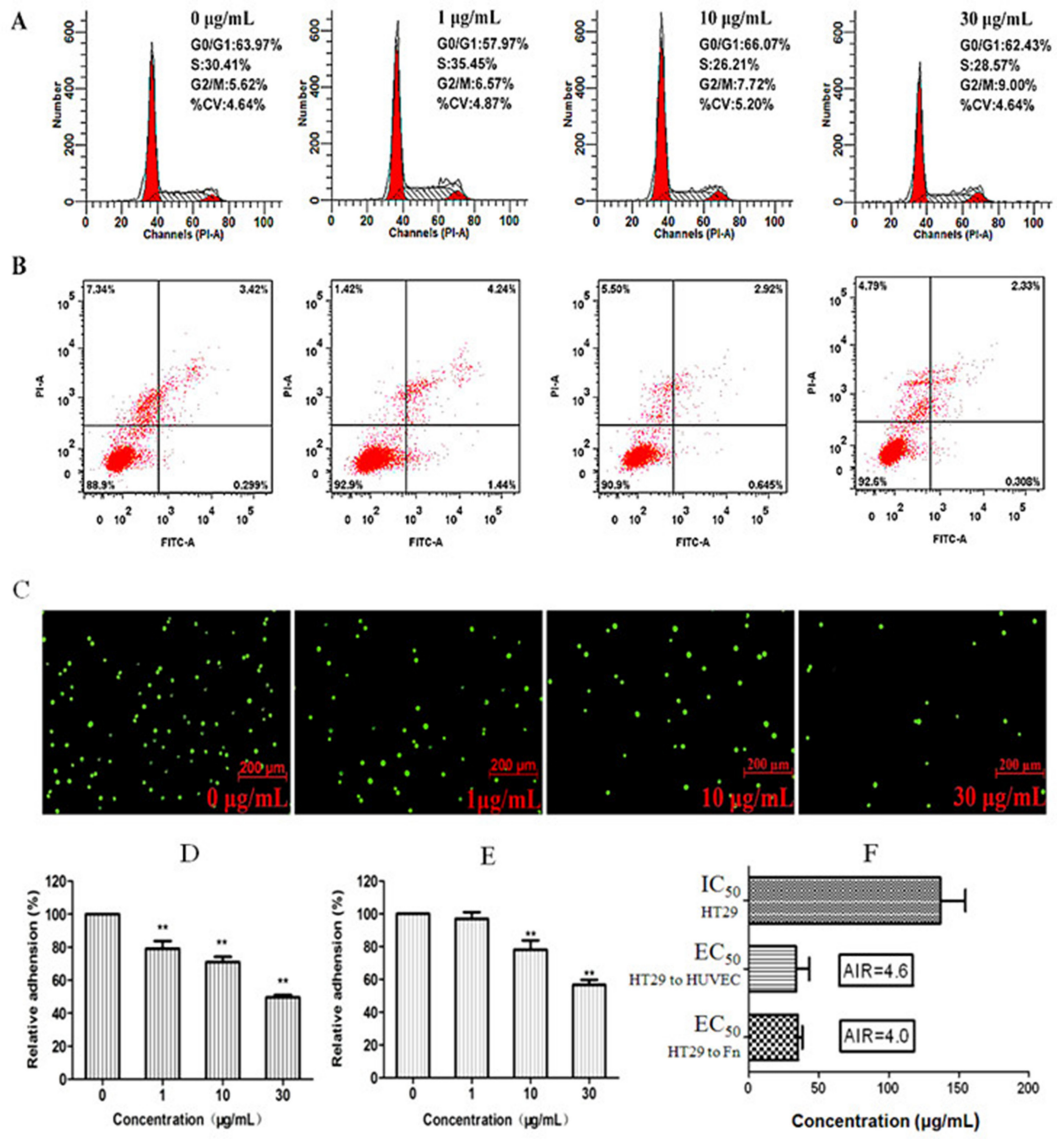
Low cytotoxicity of fraction G and its concentration-dependent (0, 1, 10, 30 μg/mL) inhibition on adhesion of cancer cell HT29 to human HUVECs and Fn-coated matrix There was no significant effect of G on HT29 cell cycle distribution (**A**) and apoptosis (**B**). However, G produced dose-dependent inhibition on Rhodamine 123-labeled HT29 cells adhered to the HUVEC monolayer stimulated by IL-1*β* (1 ng/mL) (**C**). (**D**) Quantitative inhibition of G on adhesion between HT29 cells and HUVEC monolayer. (**E)** Quantitative inhibition of G on adhesion of HT29 cells to Fn-coated matrix. (**F)** AIR indicated that G specifically inhibited hetero-adhesion between HT29 cells and HUVECs or Fn-coated matrix; the larger the AIR is, the more likely the drug works as an adhesion inhibitor. **P < 0.01, compared to the control.

G dose-dependently inhibited viability of cancer cells HT29 from 1 to 200 μg/mL after 24-h treatment with the IC_50_ around 145 μg/mL. At concentrations < 30 μg/ mL, G did not significantly affect cell cycle distribution (Figure 4A). The percent of cells treated with G and stayed at G_0_/G_1_ and S phases was close to those of the control. G produced no marked apoptosis (Figure 4B), which is usually regarded as the disqualification of a candidate for the anticancer drug. However, at low concentrations (1–10 μg/mL), G significantly inhibited adhesion of HT29 to human endothelial cells as well as to the Fn-coated matrix (Figure 4C–4E). To evaluate the specificity of G in inhibiting cellular adhesion, we created AIR to quantitatively characterize a cancer metastasis chemopreventive that is different from cytotoxic anticancer drugs in the mechanism by which a cancer metastasis chemopreventive inhibits adhesion of cancer cells to endothelial cells, instead of killing cells. The radio is calculated by dividing IC_50_ by EC_50_. The larger the AIR is, the more likely the drug works as a cancer metastasis chemopreventive by inhibiting the hetero-adhesion, rather than as a cytotoxic drug that inhibits the cellular hetero-adhesion by cell killing. As shown in Figure 4F, the AIR value of G was 4.6 (against adhesion to HUVECs), and 4.0 (against adhesion to Fn-coated matrix), respectively, suggesting the specificity of G at inhibiting cancer cell adhesion to endothelium.

### Effects of G on cancer cell migration and invasion

**Figure 5:**
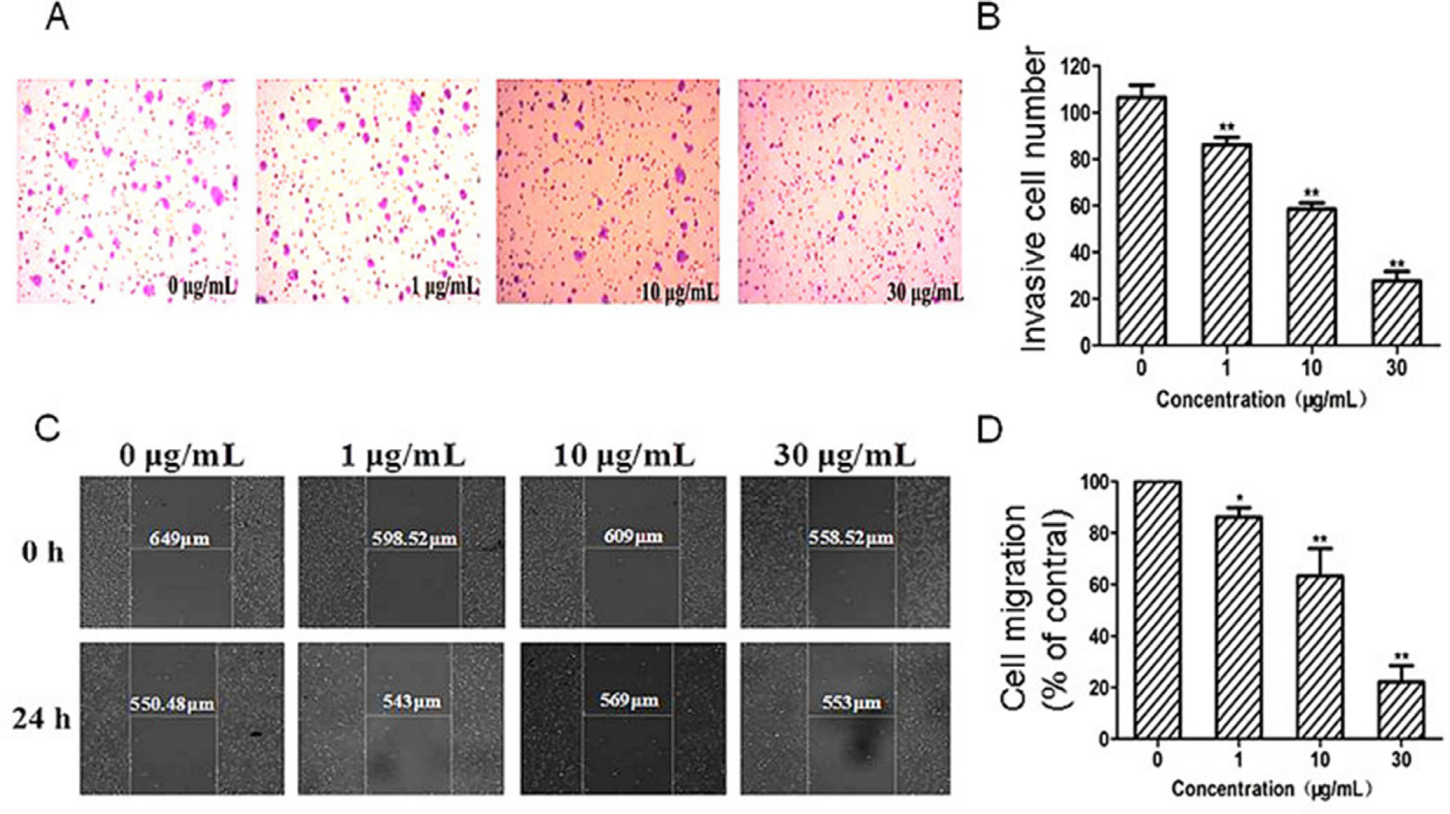
Effects of G on mobility and invasion capacity of HT29 cells (**A**) Representative images of the transwell invasion of HT29 cells treated with G (0, 1, 10 and 30 μg/mL) for 24 h. (**B)** Quantitative analysis of the effect of G on cell invasion. (**C)** Representative images of the scratch mobility of HT29 cells treated with G (0, 1, 10, 30 μg/mL) for 24 h. (**D)** Quantitative analysis of the effect of G on cell mobility. *P < 0.05; and **P < 0.01, compared with the control.

The migration and invasion ability is necessary for cancer cells to invade and extravasate through microvascular endothelium. The transwell invasion assay showed that G dose-dependently inhibited cancer cells passing through the chamber matrigel in the concentration range of 1–30 μg/mL (Figure 5A). As shown in Figure 5B, when the concentrations of G were 1, 10 and 30 μg/mL, the number of cells passed through the transwell were 81.1%, 54.7% and 26.4%, respectively,and all shown significant inhibition in comparison with that of the control (*P* < 0.01).

The scratch assay showed that G inhibited cell migration in a concentration-dependent manner (Figure 5C–5D). The migration ability of cancer cells was reduced by G treatment for 24 h. At relative low concentrations (10–30 μg/mL), G could significantly inhibited the migration ability of cancer cells in comparison with the control (*P* < 0.01; Figure 5D).

### Down-regulation on adhesion molecules in cancer cells and endothelial cells by G

**Figure 6:**
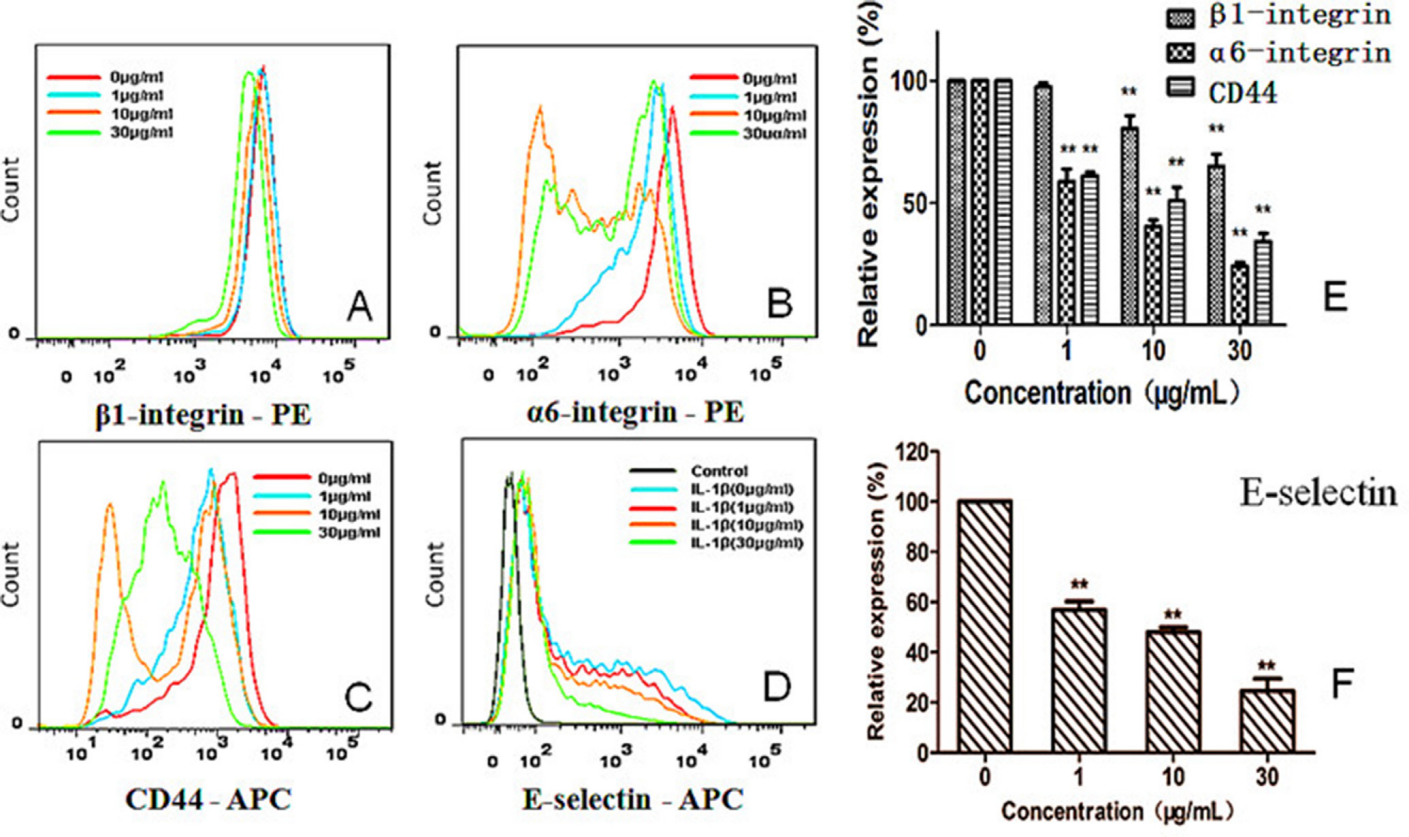
Concentration-dependent effect of G on expression of integrin *β*1 (CD29), integrin *α*6 (CD49f), and CD44 on HT29 cells (A–C, E), as well as expression of E-selectin (CD62E) on HUVECs (D, F) **P < 0.01, compared with the control

To explore what adhesion molecules were inhibited by G, we first tested inhibition by G on a variety of cellular adhesion molecules (CAMs) [8, 12]. Among those tested, integrin *β*1 (CD29) and *α*6 (CD49f) were the most significantly-affected CAMs (Figure 6A–6B). Integrin *β*1 expression was down-regulated from 97.52 ± 1.64% (1 μg/mL) and 80.40 ± 5.42% (10 μg/mL) to 65.03 ± 5.03% (30 μg/mL), respectively, by G in comparison with the control (*P* < 0.01). The expression level of integrin *α*6 (CD49f) was also down-regulated from 58.74 ± 5.20% (1 μg/mL), 40.42 ± 2.64% (10 μg/mL), and 24.36 ± 1.49% (30 μg/mL), respectively, by G, in comparison with the control (*P* < 0.01; Figure 6E). CD44 plays an important role in hetero-adhesion and promotes the invasion and metastasis of tumor cells. CD44 on HT29 cells was prominently suppressed by G in a concentration-dependent manner (Figure 6C and 6E). The CD44 expression level was down-regulated by G by 61.30 ± 1.46% (1 μg/mL), 50.89 ± 5.53% (10 μg/mL), 34.36 ± 3.27% (30 μg/mL), respectively, significantly different from that of the control (*P* < 0.01).

After stimulating HUVECs with IL-1*β* (1 ng/mL), G produced concentration-dependent inhibition on E-selectin expression by HUVECs, and the inhibition was statistically significant in comparison with the control (*P* < 0.01; Figure 6D, 6F). However, the expression of ICAM-1 by HUVECs was not notably influenced by G (data not shown).

### Chemopreventive effect of G on melanoma lung metastasis

**Figure 7:**
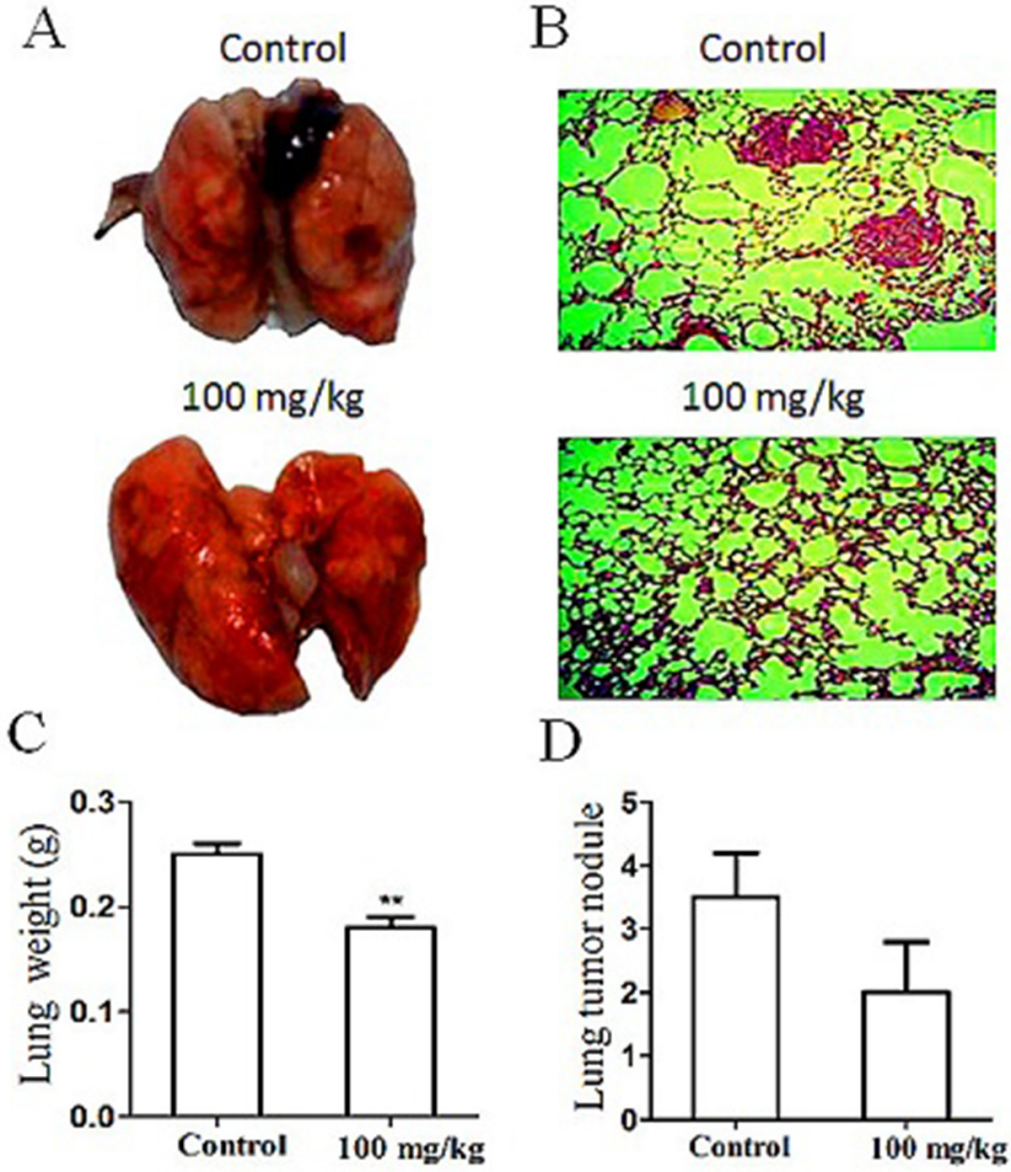
Effect of oral G (100 mg/kg/day for 30 days) on development of lung metastasis induced by mouse B16-F10 melanoma cells inoculated (*iv*) into the immunocompetent mice Images of lung metastasis (**A**) and typical lung histology (**B**) in the control (up) and experimental (down) groups (*n* = 10/group). Effect of G on mouse lung weight (**C**) and tumor nodule number (**D**). **P < 0.01, compared with the control.

HT29 cell line is derived from humans and can only be implanted into the immunodeficient nude mice as an experimental model. To observe the effects of a metastasis chemopreventive on metastatic development under the normal immunosurveillance, we used the lung metastatic experimental model as we described previously [12]. Briefly, mouse B16-F10 melanoma cells (3 × 10^5^ per mouse) were injected into C57BL/6 mice to simulate the CTCs circulating in blood. The amount of cells injected was minimal but ensured the success of lung metastasis within about 40 days. Preventive administration of oral G was conducted 4 days before the cell injection, and then G was administered for 31 days (100 mg/kg/day). As shown in Figure 7, the tumor volume and nodule number of metastatic melanoma to the lung were significantly reduced compared with those of the control. The lung histomorphological analysis revealed the least micrometastasis in lungs of the G-treated group (Figure 7A, 7B). The lung weight and melanoma nodules in lungs of G-treated mice were significantly lesser than those in the untreated control (Figure 7C, 7D). In contrast to the control, lungs of the G-treated mice showed no obvious necrosis. The result indicated the significant metastatic chemopreventive effect of G on the animal model.

## DISCUSSION

Tumors of solid organs (carcinomas, sarcomas, and central nervous system tumors) kill patients mainly by dissemination from the primary site to various tissues. The precision surgery and radiology treatments have made localized cancers largely manageable, if not curable. However, once the CTCs metastasize beyond the primary site into adjacent or distant tissue, the CTCs are very difficult, if not impossible, to control and extirpate [36], and the death becomes irreversible no matter what kind of chemotherapy is used. To revolutionize the current expensive and late post-metastasis chemotherapies, we are discovering and developing safe, inexpensive and early pre-metastasis chemopreventives [8–12]. Based on the biological similarities between embryo implantation to endometrium and CTCs adhesion-invasion to endothelium, the present study tried, for the first time, to find the safe and efficient pre-metastatic chemopreventive from the abortion TCM.

Completely different from the traditional chemotherapy strategy that aims to find cytotoxic agents or target therapeutic agents to kill cancer cells after their dissemination to various tissues, our strategy focuses on interfering with the early stage of the CTC activation-adhesion-invasion-extravasation metastasis cascade, specifically, the cascade origin, i.e., activation-adhesion point. Hypothetically, if the CTCs fail to adhere and invade the endothelial layer of distant metastatic tissues, they may die due to the loss of matrix-derived survival signals, circulatory shear stress, and/or anoikis [37]. Many safe abortion TCMs may possess the ability of interfering with the early stage of the metastasis cascade, and therefore can be repurposed for the cancer metastasis chemopreventive for the asymptomatic cancer survivors.

Many TCMs are used as the raw extract, which is indeed clinically effective if the quality of the extract can be controlled. We therefore in the present study tested the metastasis chemopreventive effect of *M. paniculata* by using the plant's raw extract, *i.e.*, fraction G, although we are currently trying to further separate each and every single component from G, and test their effects individually. The present study demonstrated that G contains primarily flavonoids and coumarins, and the two major classes of chemicals (one of which is flavonoid derivative HMFRR) may synergistically make G effective in scavenging free radicals (Figure 2C), and other bioactivities. G specifically inhibited both embryo implantation to human endometrial bed (Figure 3) and cancer HT29 cells to human endothelium (Figure 4) in a concentration-dependent manner within 1–30 μg/mL, and the inhibition concentration was far lesser than its anti-proliferative IC_50_ 145 μg/mL (Figure 1C). As a result, G demonstrated its high adhesion inhibition ratio, suggesting its specificity in inhibiting adhesion-invasion process of CTCs with a good safety margin.

Embryo implantation is a multistep process that begins with embryo apposition, followed by adhesion onto endometrial epithelial cells, penetration, and invasion into endometrial stromal cells [26]. During embryonal outgrowth (Figure 3), endometrial epithelial layer may create space for embryonic penetration. In the embryonic invasion step, the disrupted endometrial epithelial sheet is reconstructed partially by endometrial epithelial proliferation as well as motility. The embryo is then covered with the epithelial cells and the immunologic barrier restored. Up-regulation of endometrial epithelial motility is induced by steroid hormones and implantation-mediated CAMs. In the present study, we demonstrated that, by using the invasion and motility assays [38], that G inhibited invasion and migration of cancer cells at concentrations (1–30 μg/mL; Figure 5) far below its anti-proliferative IC_50_. In addition, the inhibition by G on the cellular hetero-adhesion may result from its specific down-regulation on expression of integrin *β*1 and *α*6, and CD44 on HT29 cells, as well as E-selectin on endothelial cells (Figure 6). Integrins are a large family of CAMs, which support and regulate the functions of various cells where tumor metastasis occurs as well as affect metastatic potency. Meanwhile, integrins influence the mechanisms of tumor metastasis and are involved in the adhesion of tumor cells. Moreover, they are closely associated with proteolytic enzyme and tumor angiogenesis. In the present study, we also demonstrated that G significantly inhibited the expression of integrin *β*1 and *α*6, and CD44 on HT29 cell surface in a concentration-dependent manner within the concentration range from 1 to 30 μg/mL.

Four-day pretreatment followed by one-month oral administration of G to the immunocompetent mice inoculated with mouse melanoma cells produced significant inhibition on lung metastasis without marked side effects (Figure 7). This *in vivo* experiment further demonstrated the clinical applicability of G as the cancer metastasis chemopreventive for asymptomatic cancer survivors in terms of its safety and effectiveness. Although this paradigm-shifting study provides, for the first time, a new strategy to discover the safe and effective pre-metastatic chemopreventives from abortion TCM, there is still a long way for us to go to develop these cancer metastasis chemopreventives into market to meet the urgent need of the daily-increasing cancer survivors. One of the bottle-neck is the lack of the established regulatory system to evaluate this class of drugs for asymptomatic cancer survivors and also for the corresponding health insurance policymakers. Nonetheless, the present paradigm-shifting idea and its practice call for the global attention to the long-term neglected and ignored area, that is cancer metastasis chemoprevention and related products.

## MATERIALS AND METHODS

### Reagents

Human interleukin-1 beta (IL-1*β*) was purchased from Cell Signaling Technology Inc. Mouse anti-human CD29 (Integrin *β*1)-PE, mouse anti-human CD44-APC, rat anti-human CD49f (Integrin *α*6)-PE, anti-Human CD54 (ICAM-1)-PE and mouse anti-human CD62E (E-selectin)-APC antibodies were all obtained from Becton Dickinson (BD) Pharmingen. Fluorescent dyes propidium iodide (PI) and DiOC6 were obtained from Sigma-Aldrich. Calcein-AM was obtained from Santa Cruz Biotechnology.

### Plant materials and extraction procedures

Guided by the Chinese Pharmacopoeia (2010 Ed.), we identified *Murraya paniculata (L.) Jack* which belongs to the genus *Murraya* by examining the plant's jagged-edge green leaves, bud, red fruit, as well as its white and sweet flower in summer (Figure 1A). The twigs were pieced and grounded to powders for following ethanol extraction procedure: about 4.2 kg of dried and powder twigs was extracted 3 times (3 h each time) with 5 L of 75% ethanol under reflux. The extract was pooled together and rotary-evaporated to give a syrupy extract, which was followed by ultrasonically suspension in water, and then extracted with dichloromethane four times. The dichloromethane layer was removed by rotary-evaporation. The resultant residue (40 g) was fractioned by ethyl acetate-diethyl ether gradient chromatography on a silica gel column (45 cm × 5 cm). The elution procedure, petroleum ether/EtOAc at 8: 1 (1000 mL), 7: 1 (950 mL), 6: 1 (2900 mL), 5: 1 (2330 mL), 4: 1 (1960 mL), 3: 1 (2780 mL), 2: 1 (3400 mL), 1: 1 (4380 mL) and finally EtOAc (4700 mL), gave 8 pooled fractions (A to H) depending on TLC, which were rotary-evaporated, respectively.

### Phytochemical color tests for possible constituents existed in fraction G

The existance of flavonoids in fraction G was tesed by Shinoda's test, aqueous NaOH test and concentrated H_2_SO_4_ test. For Shinoda's test, magnesium powders were added to the methanol solution of G, followed by adding of hydrochloric acid drop wisely and heating. Appearance of magenta color indicates the existence of flavonoids. For aqueous NaOH test, aqueous NaOH was added to the ethanol aqueous solution of G appearance of yellow color indicates the presence of flavonoids. For conc. H_2_SO_4_ test, concentrated H_2_SO_4_ was added to the ethanol solution of G, and orange color indicates the presence of fla vonoids.

The possible coumarins were tested by fluorescence detection and dilute ferric chloride solution. Fraction G was loaded on a thin layer silica gel plate, under the irradiation of 365 nm, appearance of blue fluorescence and enhanced green fluorescence with spraying of 10% sodium hydroxide solution indicates the presence of coumarins. As to the ferric chloride test, FeCl_3_ solution (5% w/v) was added to small amount of fraction G's methanol aqueous solution, and the appearance of deep green color indicates t presence he of coumarins.

### HPLC separations of fraction G

To separate and isolate the compositions of fraction G, we developed the HPLC analysis and semi-preparation methods similar to what we reported previously [15]. Briefly, the HPLC system was composed of Waters 2695 pump with Waters 2475 fluorescence detector and Waters 2489 UV-Vis detector. The analytic HPLC was performed on an Waters Sunfire C18 column (15 cm × 4.6 mm, 5 μm) maintained at 35°C with isocratic elution of methanol-water (45: 55, v: v) at the flow rate of 1.0 mL/min. The injection volume was 20 μL and the UV-Vis detection wavelength was 280 nm. Semi-preparative HPLC was performed on an Agela Venusil XBP C18 column (25 cm× 10 mm, 5 μm) maintained at 35°C. The elution program was the same except the flow rate was 3.0 mL/min. The injection volume was 200 μL. The fluorescence detection wavelength was set as: λ_ex_ = 352 nm, λ_em_ = 458 nm. The corresponding HPLC peak was collected, followed by rotary evaporation and lyophilization, giving amorphous powders.

### DPPH radical-scavenging assay of fraction G in comparison with ascorbic acid

The radical scavenging activity of fraction G was determined based on the rapid 2,2-diphenyl-1-picrylhydrazyl (DPPH) test in comparison with the common antioxidant ascorbic acid. Briefly, 0.1 mmol/L DPPH solution in ethanol (100 μL per well) and the sample solution in ethanol (100 μL per well) was added in the 96-well plates. Ethanol was used as a blank solution. DPPH solution (100 μL) with additional methanol (100 μL) served as the control. The concentration of the fraction G or ascorbic acid samples were 10, 20, 60, 125, 250, 500 μg/mL. After standing at room temperature in the dark for 30 min, the decrease of DPPH radical absorbance at 517 nm was measured and the radical scavenging rate was calculated as following: Radical scavenging rate (%) = [1− (A _sample_−A _blank_)/A _control_] × 100%

### Cell lines and cell culture conditions

Human endometrial cell line RL95-2 was bought from Tongpai Science and Technology Co., Ltd. Human velvet JEG-3 cell line was a gift from Maternity and Child Care Centers of Fujian Province and was used as a model for blastocysts. Human colorectal cancer cell line HT29 and melanoma cancer cell line B16-F10 were obtained from the Cell Bank of Type Culture Collection of Chinese Academy of Sciences. The cells were cultured respectively in RPMI 1640 (RL95-2), DMEM (JEG-3) or McCOY's 5A medium (B16-F10) supplemented with 10% fetal bovine serum, 100 units/mL penicillin and 100 mg/mL streptomycin.

Human umbilical vein endothelial cells (HUVECs) were separated and cultured as we described previously [8]. The cells were maintained in 1% gelatin-coated tissue culture flasks in M199 (Gibco) medium supplemented with 20% FBS, 8 units/mL heparin, 100 mg/mL ECGS, 100 units/mL penicillin and 100 mg/mL streptomycin. HUVECs were not used after 6 passages.

All these cells were cultured at 37°C in a humid atmosphere with 5% CO_2_.

### Mice

C57BL/6 mice (20 ± 2 g, 6–8 weeks old) were purchased from Shanghai SLAC Laboratory. These mice were maintained with free access to pellet food and water in a controlled environment of 20–25°C and 12-h light/ dark circles. All animals used in the investigation were handled in accordance with the Guide for the Care and Use of Laboratory Animals (National Research Council, 1996), and approved by the institutional animal care and use committee of Fuzhou University.

### Spheroid adhesion assay

RL95-2 cells were seeded into 96-well plates, 5 × 10^4^ cells per well, and cultured for 24 h to form monolayer followed by another 24 h of treatment of G from 0 to 60 μg/mL. Trophoblast spheroids were generated by shaking JEG-3 cells on a gyratory shaker at 90 rpm for 24 h. At the end of spheroid preparation, calcein-AM fluorescent dye was added to the suspension and incubated for 30 min. JEG-3 spheroids (*n* = 50–60) were delivered to each well with a confluent monolayer of RL95-2 cells, and incubated for 1 h. Adhered spheroids were counted under a fluorescent microscope and adhesion rates were calculated by the formula:

Adhesion rates (%) = Number of adhered spheroids/number of total spheroids delivered × 100%.

### Cytotoxicity assay

Cell viability was assessed using MTT assay as we described previously (8, 39). In brief, HT29 cells (8 × 10^4^−1 × 10^5^ per well) or HUVECs (10^4^ per well) were cultivated in 96-well plates for 24 h and then incubated with various concentrations of fraction G (1–200 μg/mL) for 24 h before the 4-h MTT assay. The results were read at OD_492__nm_ on an ELISA reader. The percent viability of cells exposed to treatments was expressed as ratio to that of the untreated control as follows:

Relative inhibition rate (%) = [1 - (A-A_0_) / (A_1_-A_0_)] × 100%

A: OD value of the experimental group; A_0_: OD value of the blank control group; A_1_: OD value of the parallel solvent control group.

### Cell cycle analysis

Cell cycle was analyzed by flow cytometry for DNA content as we described previously [9]. The HT29 cell activity expressed as cell cycle distribution was determined by flow cytometry after incubation with or without G (0, 1, 10 and 30 μg/mL) for 24 h. The cells were washed with ice-cold PBS, fixed in 80% ice-cold ethanol overnight, and then spun to remove ethanol before cellular DNA staining with the fluorescent solution (1% Triton X-100, 0.01% RNase, 0.05% PI) and incubated at room temperature in dark for 45 min. Cell cycle was assessed by using the FACSAriaIII flow cytometer and analyzed by Modfit software.

### Flow cytometry

Flow cytometric analysis of CAMs expression on cells was performed on a BD FACSAriaIII cell sorter with laser excitation set at 488 and 633 nm, as we described previously [*8*]. BD FACSDiva software provided with the system was employed for data acquisition and initial data analysis. Forward versus side scatter histograms were utilized to gate on single intact cells. The data were collected in FCS format with the subsequent analysis based on 10, 000 cells to meet the light scatter criteria. PE and PI dyes were excited by 488 nm laser and detected through 585 and 530 nm bandpass filters. APC dye was excited by 633 nm laser, and derived fluorescence was detected through 660 nm bandpass filter. FITC dye was excited by 488 nm and detected through 530 nm bandpass filter.

### Adhesion assay of HT29 to HUVEC and Fn-coated matrix

Quantification of HT29 cancer cell heter-adhesion to endothelial cells or Fn was carried out as we described previously [8, 12]. Quantification of HT29 cancer cell adhesion to endothelial cells was carried out as we described previously [8, 12]. HUVECs were seeded on a 24-well plate and cultured to form confluent cell monolayer, followed by pretreatment of 500 μL of 1 ng/mL IL-1*β* for 4 h. In the meantime, HT29 cells were mixed with Rhodamine 6G protecting from light at room temperature for 15 min. Then the cells were re-suspended in RPMI 1640 media with indicated concentrations of G, and added to the wells covered with HUVECs, 1.5 × 10^5^ per well. After another 1 h of incubation, the unadhered cells were gently washed off with PBS twice. Fluorescence-labeled HT29 cells were quantified by an inverted fluorescence microscope (Zeiss, Germany). The mean inhibition of adhesion for 10 visual fields was calculated as follows:

Relative adhesion% = Number of adhered cells of experimental group/number of adhered cells of control group × 100%

MTT-based analysis was used to evaluate effects of fraction G on the hetero-adhesion of HT29 to the Fn, the later was used to simulate the extracellular matrix. Firstly, the 96-well plate was coated with 100 μL 10 μg/mL Fn per well and incubated for 24 h. Secondly, the Fn solution was replaced with 1% BSA, 100 μL per well. After 1 h incubation, the BSA was removed and washed thoroughly with PBS for three times. Thirdly, HT29 cells (2 × 10^4^/well) were seeded on as-treated plate and with different concentration of fraction G (0, 1, 10, 30 μg/mL) for 1 h. Then, non-adhered HT-29 cells were removed by PBS washing for three times. The relative rate of adhesion of HT29 to Fn effected by G was determined by the MTT assay and calculated by the following equation:

Relative adhesion rate (%) = (OD value of treatment group- solvent blank group OD value)/(control group OD value- solvent blank group OD value) × 100%.

### Scratch assay

Scratch assay was performed to analyze cell migration *in vitro*, as described in our previous work [19]. Briefly, HT29 cells were seeded in 24-well plates and incubated to form confluent. Sterile tips were used to scratch cell layers, which were subsequently washed with PBS for three times, and then cultured with 1 mL of DMEM media containing 2% FBS and different concentrations (1, 10, 30 μg/mL) of G. The cells were photographed (phase-contrast microscope) at 0 and 24 h. The distance travelled by cells was measured between the two boundaries of an acellular area, and results of treatment groups were expressed as ratios to that of the control cells (without addition of G). Each experiment was performed in triplicate.

### Tumor cell invasion assay

Inhibition capacity against invasion of HT29 cells was estimated by transwell invasion assay as we described previously [19]. The upper chambers of the transwells (24-well, 8 μm pore size, Costar, Corning Incorporated, USA) were treated with Matrigel (Becton Dickinson, Waltham, MA, USA) and air-dried. The lower chambers were added with 750 μL of media containing 20% FBS. HT29 cells were seeded at the density of 3×10^4^ per well (200 μL) into the upper chambers in DMEM containing determined concentrations of G and 0.1% BSA. After 24 h, the cells that had invaded through the Matrigel membrane were stained with crystal violet, and counted (five random fields).

### Development of lung metastasis induced by B16-F10 melanoma cells

Fraction G was suspended in 0.5% carboy methyl cellulose-Na. Twelve female C57BL/6 mice (6 to 8-weeks- old) were randomly divided into control and treated groups. The chemopreventive effect of fraction G (4-day oral pretreatment of 100 mg/kg before B16-F10 inoculation, followed by a 30- day treatment) was determined on C57BL/6 mice inoculated with minimal metastatic B16-F10 melanoma cells (3 × 10^4^ / 0.2 mL/ mice). One-month after inoculation of the tumor cells, the mice were sacrificed and their lungs were excised. The number of surface melanoma colonies was counted under a dissecting microscope. The lungs of each mouse were weighed respectively and prepared into tissue sections for histological observation.

### Statistical analysis

Data were analyzed using SPSS statistics 17.00 and presented as means ± S.D. The difference between experimental and control groups was determined by Student's *t* test. A *P*-value of less than 0.05 was considered statistically significant, and less than 0.01 to be extremely statistically significant.

## References

[1] Alix-Panabieres C, Pantel K (2014). OPINION Challenges in circulating tumour cell research. Nat Rev Cancer.

[2] de Moor JS, Mariotto AB, Parry C, Alfano CM, Padgett L, Kent EE, Forsythe L, Scoppa S, Hachey M, Rowland JH (2013). Cancer survivors in the United States: prevalence across the survivorship trajectory and implications for care. Cancer Epidemiol Biomarkers Prev.

[3] DeSantis CE, Lin CC, Mariotto AB, Siegel RL, Stein KD, Kramer JL, Alteri R, Robbins AS, Jemal A (2014). Cancer treatment and survivorship statistics, 2014. CA Cancer J Clin.

[4] Perret GY (2010). Pharmacological strategies and micrometastasis: what is known?. What must be done? Minerva Med.

[5] Quail DF, Joyce JA (2013). Microenvironmental regulation of tumor progression and metastasis. Nat Med.

[6] Valastyan S, Weinberg RA (2011). Tumor metastasis: molecular insights and evolving paradigms. Cell.

[7] Yokota J (2000). Tumor progression and metastasis. Carcinogenesis.

[8] Lu YS, Yu T, Liang HY, Wang JC, Xie JJ, Shao JW, Gao Y, Yu SH, Chen S, Wang L, Jia L (2014). Nitric oxide inhibits hetero-adhesion of cancer cells to endothelial cells: restraining circulating tumor cells from initiating metastatic cascade. Sci Rep.

[9] Wang JC, Chen JZ, Wan LY, Shao JW, Lu YS, Zhu YW, Ou MR, Yu SH, Chen HJ, Jia L (2014). Synthesis, spectral characterization, and *in vitro* cellular activities of metapristone, a potential cancer metastatic chemopreventive agent derived from mifepristone (RU486). AAPS J.

[10] Xie JJ, Lu YS, Dong HY, Zhao RL, Chen HN, Shen WY, Sinko PJ, Zhu YW, Wang JC, Shao JW, Gao Y, Xie FW, Jia L (2015). Enhanced Specificity in Capturing and Restraining Circulating Tumor Cells with Dual Antibody-Dendrimer Conjugates. Adv Funct Mater.

[11] Xie JJ, Zhao RL, Gu SG, Dong HY, Wang JC, Lu YS, Sinko PJ, Yu T, Xie FW, Wang L, Shao JW, Jia L (2014). The Architecture and Biological Function of Dual Antibody-Coated Dendrimers: Enhanced Control of Circulating Tumor cells and Their Hetero-Adhesion to Endothelial Cells for Metastasis Prevention. Theranostics.

[12] Wan L, Dong H, Xu H, Ma J, Zhu Y, Lu Y, Wang J, Zhang T, Li T, Xie J, Xu B, Xie F, Gao Y (2015). Aspirin, lysine, mifepristone and doxycycline combined can effectively and safely prevent and treat cancer metastasis: prevent seeds from gemmating on soil. Oncotarget.

[13] Leong SP, Tseng WW (2014). Micrometastatic cancer cells in lymph nodes, bone marrow, and blood: Clinical significance and biologic implications. CA Cancer J Clin.

[14] Lu Y, Liang H, Yu T, Xie J, Chen S, Dong H, Sinko PJ, Lian S, Xu J, Wang J, Yu S, Shao J, Yuan B (2015). Isolation and characterization of living circulating tumor cells in patients by immunomagnetic negative enrichment coupled with flow cytometry. Cancer.

[15] Xie J, Dong H, Chen H, Zhao R, Sinko PJ, Shen W, Wang J, Lu Y, Yang X, Xie F, Jia L (2015). Exploring cancer metastasis prevention strategy: interrupting adhesion of cancer cells to vascular endothelia of potential metastatic tissues by antibody-coated nanomaterial. J Nanobiotechnology.

[16] Xie J, Gao Y, Zhao R, Sinko PJ, Gu S, Wang J, Li Y, Lu Y, Yu S, Wang L, Chen S, Shao J, Jia L (2015). *Ex vivo* and *in vivo* capture and deactivation of circulating tumor cells by dual-antibody-coated nanomaterials. J Control Release.

[17] Xie J, Wang J, Chen H, Shen W, Sinko PJ, Dong H, Zhao R, Lu Y, Zhu Y, Jia L (2015). Multivalent conjugation of antibody to dendrimers for the enhanced capture and regulation on colon cancer cells. Sci Rep.

[18] Gao Y, Li Z, Xie X, Wang C, You J, Mo F, Jin B, Chen J, Shao J, Chen H, Jia L (2015). Dendrimeric anticancer prodrugs for targeted delivery of ursolic acid to folate receptor-expressing cancer cells: synthesis and biological evaluation. Eur J Pharm Sci.

[19] Jiang Z, Yang JN, Pang YQ, Yang XT, Yu SH, Jia L (2015). Bioactivity-guided fast screen and identification of cancer metastasis chemopreventive components from raw extracts of *Murraya exotica*. J Pharmaceut Biomed.

[20] Xiang L, Chi T, Tang Q, Yang X, Ou M, Chen X, Yu X, Chen J, Ho RJ, Shao J, Jia L (2015). A pentacyclic triterpene natural product, ursolic acid and its prodrug US597 inhibit targets within cell adhesion pathway and prevent cancer metastasis. Oncotarget.

[21] Beral V, Doll R, Hermon C, Peto R, Reeves G (2008). Ovarian cancer and oral contraceptives: collaborative reanalysis of data from 45 epidemiological studies including 23,257 women with ovarian cancer and 87,303 controls. Lancet.

[22] Hannaford PC, Iversen L, Macfarlane TV, Elliott AM, Angus V, Lee AJ (2010). Mortality among contraceptive pill users: cohort evidence from Royal College of General Practitioners' Oral Contraception Study. BMJ.

[23] Yu XB, Shi Q, Liu J, Lu YS, Chi T, Liu YJ, Shao JW, Jia L Molecular and cellular similarities and differences between embryo implantation and cancer cell adhesion-invasion.

[24] Chen J, Wang J, Shao J, Gao Y, Xu J, Yu S, Liu Z, Jia L (2014). The unique pharmacological characteristics of mifepristone (RU486): from terminating pregnancy to preventing cancer metastasis. Med Res Rev.

[25] Yu S, Yang X, Zhu Y, Xie F, Lu Y, Yu T, Yan C, Shao J, Gao Y, Mo F, Cai G, Sinko PJ, Jia L (2015). Systems pharmacology of mifepristone (RU486) reveals its 47 hub targets and network: comprehensive analysis and pharmacological focus on FAK-Src-Paxillin complex. Sci Rep.

[26] Uchida H, Maruyama T, Nishikawa-Uchida S, Oda H, Miyazaki K, Yamasaki A, Yoshimura Y (2012). Studies using an *in vitro* model show evidence of involvement of epithelial-mesenchymal transition of human endometrial epithelial cells in human embryo implantation. J Biol Chem.

[27] Ferretti C, Bruni L, Dangles-Marie V, Pecking AP, Bellet D (2007). Molecular circuits shared by placental and cancer cells, and their implications in the proliferative, invasive and migratory capacities of trophoblasts. Hum Reprod Update.

[28] Fukuda MN, Sugihara K (2012). Trophinin in cell adhesion and signal transduction. Front Biosci (Elite Ed).

[29] Murray MJ, Lessey BA (1999). Embryo implantation and tumor metastasis: Common pathways of invasion and angiogenesis. Semin Reprod Endocr.

[30] Pang PC, Chiu PC, Lee CL, Chang LY, Panico M, Morris HR, Haslam SM, Khoo KH, Clark GF, Yeung WS, Dell A (2011). Human sperm binding is mediated by the sialyl-Lewis(x) oligosaccharide on the zona pellucida. Science.

[31] Perry JK, Lins RJ, Lobie PE, Mitchell MD (2010). Regulation of invasive growth: similar epigenetic mechanisms underpin tumour progression and implantation in human pregnancy. Clin Sci (Lond).

[32] Menezes IR, Santana TI, Varela VJ, Saraiva RA, Matias EF, Boligon AA, Athayde ML, Coutinho HD, Costa JG, Rocha JB (2015). Chemical composition and evaluation of acute toxicological, antimicrobial and modulatory resistance of the extract of Murraya paniculata. Pharm Biol.

[33] Cuong NM, Khanh PN, Duc HV, Huong TT, Tai BH, Binh NQ, Durante M, Fusi F (2014). Vasorelaxing Activity of Two Coumarins from *Murraya paniculata* Leaves. Biological and Pharmaceutical Bulletin.

[34] Wu L, Li P, Wang X, Zhuang Z, Farzaneh F, Xu R (2010). Evaluation of anti-inflammatory and antinociceptive activities of *Murraya exotica*. Pharm Biol.

[35] Jia L, Zhao Y (2009). Current evaluation of the millennium phytomedicine- Ginseng (I): etymology, pharmacognosy, phytochemistry, market and regulations. Current medicinal chemistry.

[36] Wells A, Grahovac J, Wheeler S, Ma B, Lauffenburger D (2013). Targeting tumor cell motility as a strategy against invasion and metastasis. Trends Pharmacol Sci.

[37] Frisch SM, Francis H (1994). Disruption of epithelial cell-matrix interactions induces apoptosis. J Cell Biol.

[38] Hur K, Toiyama Y, Takahashi M, Balaguer F, Nagasaka T, Koike J, Hemmi H, Koi M, Boland CR, Goel A (2013). MicroRNA-200c modulates epithelial-to-mesenchymal transition (EMT) in human colorectal cancer metastasis. Gut.

[39] Wang J, Jiang Z, Xiang L, Li Y, Ou M, Yang X, Shao J, Lu Y, Lin L, Chen J, Dai Y, Jia L (2014). Synergism of ursolic acid derivative US597 with 2-deoxy-D-glucose to preferentially induce tumor cell death by dual-targeting of apoptosis and glycolysis. Sci Rep.

